# Emotional Mental Imagery Abnormalities in Monozygotic Twins With, at High-Risk of, and Without Affective Disorders: Present in Affected Twins in Remission but Absent in High-Risk Twins

**DOI:** 10.3389/fpsyt.2019.00801

**Published:** 2019-11-08

**Authors:** Martina Di Simplicio, Alex Lau-Zhu, Iselin Meluken, Patrick Taylor, Lars Vedel Kessing, Maj Vinberg, Emily Alexandra Holmes, Kamilla Woznica Miskowiak

**Affiliations:** ^1^Division of Psychiatry, Department of Brain Sciences, Imperial College London, London, United Kingdom; ^2^Social, Genetic and Developmental Psychiatry Centre, Institute of Psychiatry, Psychology and Neuroscience, King’s College London, London, United Kingdom; ^3^Medical Sciences Division, Oxford Institute of Clinical Psychology Training, University of Oxford, Oxford, United Kingdom; ^4^Copenhagen Affective Disorder Research Centre (CADIC), Psychiatric Centre Copenhagen, Copenhagen University Hospital, Rigshospitalet, Copenhagen, Denmark; ^5^Division of Psychology, Department for Clinical Neuroscience, Karolinska Institutet, Stockholm, Sweden; ^6^Department of Psychology, Uppsala University, Uppsala, Sweden; ^7^Department of Psychology, University of Copenhagen, Copenhagen, Denmark

**Keywords:** mental imagery, future simulation, bipolar disorder, depression, twins, endophenotype

## Abstract

**Background:** Mental imagery abnormalities feature across affective disorders including bipolar disorder (BD) and unipolar depression (UD). Maladaptive emotional imagery has been proposed as a maintenance factor for affective symptomatology and a target for mechanism-driven psychological treatment developments. Where imagery abnormalities feature beyond acute affective episodes, further opportunities for innovation arise beyond treatments, such as for tertiary/relapse prevention (e.g., in remitted individuals) or primary prevention (e.g., in non-affected but at-risk individuals). The aim of our study was to investigate for the first time the presence of possible mental imagery abnormalities in affected individuals in remission and at-risk individuals for affective disorders using a familial risk design.

**Methods:** A population-based cohort of monozygotic twins was recruited through linkage between the Danish national registries (*N*=204). Participants were grouped as: affected (remitted BD/UD; *n* = 115); high-risk (co-twin with history of BD/UD; *n* = 49), or low-risk (no co-twin history of BD/UD; *n* = 40). Twins completed mental imagery measures spanning key subjective domains (spontaneous imagery use and emotional imagery) and cognitive domains (imagery inspection and imagery manipulation).

**Results:** Affected twins in remission reported enhanced emotional mental imagery compared to both low- and high-risk twins. This was characterized by greater impact of i) intrusive prospective imagery (Impact of Future Events Scale) and ii) deliberately-generated prospective imagery of negative scenarios (Prospective Imagery Task). There were no significant differences in these key measures between affected BD and UD twins in remission. Additionally, low- and high-risk twins did not significantly differ on these emotional imagery measures. There were also no significant differences between the three groups on non-emotional measures including spontaneous imagery use and cognitive stages of imagery.

**Conclusions:** Abnormalities in emotional prospective imagery are present in monozygotic twins with affective disorders in remission—despite preserved cognitive stages of imagery—but absent in unaffected high-risk twins, and thus do not appear to index familial risk (i.e., unlikely to qualify as “endophenotypes”). Elevated emotional prospective imagery represents a promising treatment/prevention target in affective disorders.

## Introduction

Mental imagery refers to the experience of perception in the absence of external sensory input, for example “seeing in the mind’s eye” (1). Cognitive science suggests that mental imagery is more emotionally-evocative than its verbal-based counterpart (2). Emotional imagery symptoms feature across affective disorders (3–6). Clinical formulation in psychology suggests such mental imagery contributes to the disorder maintenance, and as such imagery presents opportunities for treatment innovation, especially for areas of considerable clinical challenge such as bipolar disorder/BD (7) and anhedonia in unipolar depression/UD (8). In psychopathology, adaptive positive imagery can also be promoted (9–11), and conversely, maladaptive negative imagery can be disrupted (12–15).

Imagery—in models of psychological treatments—has been primarily conceptualized as a maintenance (“proximal”) factor, i.e., keeping the disorder going once it developed. Delineating the role of imagery throughout illness progression and outside of the illness episode can help map other areas of application that stand to gain from a focus on imagery. For example, emotional imagery abnormalities, which are (causal) risk factors proceeding disorder onset, might be addressed to prevent disorder emergence (i.e., primary prevention). Likewise, imagery abnormalities that persist in remission can serve as target for keeping the individual well and preventing relapse (i.e., tertiary prevention). Importantly, identifying potential cognitive maintenance and/or risk factors paves the way for testing mechanistic hypotheses and mechanism-based interventions—both psychological and pharmacological (16).

Affective disorders (UD and BD, defined as disorders in which the fundamental disturbance is a change in affect or mood to depression) (17) hold one of the highest burdens of disease worldwide (18). There is a need for better identification of risk and resilience markers to develop better therapeutic interventions at different stages of disease. Affective disorders present variable degrees of heritability, from high rates in BD (19, 20) to moderate rates in UD (20).

Biases in cognition—in both “cold” (non-emotional) and “hot” (emotional-laden) information processing spanning domains of perception, attention, memory, and learning—have been associated with acute disorder episodes (21, 22) well as after recovery from acute episodes (23, 24) and in familial risk (25). Cognitive abnormalities that persist in remitted states (i.e. are state-independent) may represent illness-related traits, conferring cognitive vulnerability for relapse (26, 27). If trait-abnormalities also meet further criteria such as heritability and higher frequency in individuals at familial risk compared to the general population, these may constitute “endophenotypes” of the disorder—i.e., potentially lying along the causal pathway between genes and disorder (28)—which could guide the discovery of genetic etiological mechanisms and inform clinical efforts including preventative strategies targeting such mechanisms in disorders with high genetic risk.

Emotional imagery has been proposed to be an “emotional amplifier” that drives both depressive/anxiety and manic symptoms in BD (29). Such images may depict aspects of the future, thought to underline associated emotions and behaviors such as wellbeing, prediction, and planning (30, 31). BD and UD have both been associated with heightened involuntary and intrusive prospective imagery (32–34)—more recurrent and impactful images of personally-relevant future real-world scenarios that spring to mind unbidden (e.g., an upcoming job interview). BD and UD have also been associated with more vivid and “real” future negative images that are deliberately-generated, such as under direct instructions in the laboratory to imagine in response to statements such as “someone close will reject you” (32). Some phenomenological aspects may show more disorder-specific profiles. For instance, imagery can be “overactive” in positive states in BD only (32, 35) possibly driving escalation to mania similar to other positive emotion biases (36), and suicidal imagery may be more “compelling” in BD compared to UD (37) in line with higher suicidal rates in BD (38, 39).

There is a paucity of studies of cognitive (non-emotional) aspects of mental imagery in affective disorders, such as studies based on a key computational model that identifies four cognitive stages of mental imagery (1, 40). In this model, mental images are thought to be initially created from either short-term or long-term memory (i.e., generation); once held in mind temporarily avoiding immediate decay (i.e., maintenance), such images’ characteristics can be interpreted/scanned (i.e., inspection) and further transformed (i.e., manipulation). Available evidence from one study in UD points to potential deficits in both imagery generation and manipulation (41). The only study simultaneously assessing emotional and cognitive domains of imagery across affective disorders showed that in BD performance on non-emotional imagery tasks may vary depending on the cognitive stage tested, with deficits present in imagery manipulation alongside superior performance in imagery maintenance (32).

To date, imagery research on BD and UD has primarily involved individuals with mix of acute and recovered depression episodes (32, 33, 35, 37), and abnormalities remain untested for remitted states in affective disorders.

Imagery may also play a role in the etiology of affective disorders, i.e., involved also in the initial emergence of the disorder. This is supported by abnormalities detected in non-clinical samples with subclinical features of BD and UD (42–45) and UD (30, 46, 47). Nevertheless, it is unclear whether or not such imagery-based abnormalities reflect *familial* risk and represent candidate “endophenotypes” for affective disorders (28).

The present study investigated the presence of imagery-based abnormalities in a population-based cohort of monozygotic twins, grouped as affected (remitted or partially-remitted twins with personal history of BD/UD), high-risk (unaffected twins with co-twin history of BD/UD), and low-risk (unaffected twins with no co-twin history of BD/UD). Participants completed assessments of subjective domains of mental imagery and cognitive (non-emotional) imagery stages, informed by our previous research (32,40).

Our primary aims were to delineate whether imagery abnormalities i) persist in remission (by comparing affected twins in remission versus unaffected low-risk twins); and ii) are present in twins at high familial risk for affective disorders (by comparing high-risk twins versus both remitted and low-risk twins). If both i) and ii) were true, this would be consistent with the proposition that imagery abnormalities are candidate “endophenotypes” of affective disorders. Further, we conducted exploratory analyses separating BD from UD; comparing BD *vs*. UD affected twins directly; and assessing imagery-symptom links transdiagnostically.

## Method

### Participants

A nationwide record linkage of the Danish Twin Registry (48) and the Danish Psychiatric Central Research Register (DPCRR) (49) identified eligible monozygotic twins. In addition to monozygosity, eligibility criteria were i) a personal or co-twin history of an affective spectrum diagnosis (i.e. International Classification for Diseases ICD-10 codes F30-34.0 and F38.0) (17) or for low-risk twins neither a personal nor a co-twin history affective spectrum diagnosis from January 1995 to June 2014, and ii) age 18–50 years.

Exclusion criteria for all groups were: birth weight under 1.3 kg, history of brain injury; current severe somatic illness, current substance abuse; current mood episode defined by Hamilton Depression Rating Scale (HDRS-17) (50) or Young Mania Rating Scale (51) (YMRS; scores >14), current pregnancy, or being dizygotic. The low-risk twins were also excluded if they reported other first-degree relatives with organic mental disorder, schizophrenia spectrum, or affective disorders.

Participants provided their written and informed consent to the study in accordance with the Helsinki declaration. The study was approved by the ethics committee for the Capital Region of Denmark (H-3-2014-003) and the Danish data protection agency (2014–331–0751).

Recruitment took place from December 2014 until January 2017. From an initial sample of 215 participants, 11 participants were excluded due to missing diagnoses (*n* = 6), affective disorder (*n* = 4), or alternative high-risk affective disorder (*n*=1). The final sample for this paper included 204 participants, with each classified as affected (*n* = 115; BD = 31 and UD = 84), high-risk (*n* = 49; BD = 11 and UD = 38), or low-risk (*n* = 40). There were 25 concordant affected twin-pairs (BD/BD: *n* = 5; UD/UD: *n* = 11; BD/UD: *n* = 9), 45 discordant twin-pairs (high-risk/UD: *n* = 36; high-risk/BD: *n* = 9), 19 low-risk twin pairs, and 26 single twins.

### Overall Procedure

Participants attended a 1-day assessment at the Danish Research Centre for Magnetic Resonance at Copenhagen University Hospital Hvidovre. Further data from this sample have been reported elsewhere, including neurocognitive (52–55), clinical/psychological (56), and biological outcomes (57). Here we report data on assessments related to mental imagery for the first time.

### Assessments

#### Clinical Characteristics

Diagnoses of psychiatric illness were assessed with the Schedules for Clinical Assessment in Neuropsychiatry (SCAN) (58). Ratings and a SCAN interview were conducted by two PhD students blinded to the DPCRR register diagnoses. The research diagnoses obtained from the SCAN interviews determined the final assignment to groups. Pre-morbid intelligence (IQ) was assessed with the Danish Adult Reading Task (59). Depressive symptoms were assessed with the HDRS-17 (50). State anxiety was assessed with the State-Trait Anxiety Inventory form-Y (STAI-Y) (60). Manic symptoms were assessed with the YMRS (51).

#### Subjective Domains of Mental Imagery

##### Spontaneous Imagery Use

The Spontaneous Use of Imagery Scale (SUIS) (61) is a 12-item self-report scale which measures the use of non-emotional mental imagery in daily life. Each item is rated on a 5-point Likert-type scale. Total scores range from 12 to 60, with higher scores indicating greater use of imagery. An example item is “When I think about a series of errands I must do, I visualize the stores I will visit”.

##### Emotional Mental Imagery

Three assessments were considered to gauge various aspects of emotional imagery. The *Impact of Future Events Scale (IFES)* (62) is a 24-item self-report scale which is used to assess intrusive (involuntary) future-oriented imagery. Participants were asked to identify three future events which they had thought about or imagined over the previous week, and state whether each was positive or negative. Participants then responded to 24 statements about prospective imagery in relation to these events. Each item is rated on a 5-point Likert-type scale. Total scores range from 0 to 96, with higher scores indicating greater emotional impact of *involuntarily-generated* prospective imagery.

The *Prospective Imagery Task (PIT)* (63) is used to assess aspects of deliberately-generated (voluntary) imagery for imagined future events. Participants were presented with 10 positive and 10 negative hypothetical future scenarios and asked to generate a mental image of each. They were then asked to rate each image of a 5-point Likert-type scale, assessing vividness, likelihood of the event occurring to them, and how much they felt as though they were experiencing the event while imagining it. Total scores for each dimension range from 20 to 100, with higher total scores indicating greater subjective experience of *deliberately-generated* prospective imagery.

The *Mental Imagery Interview (MII)* (32) is a semi-structured interview which contains 12-items each for low-mood, anxious, and high-mood states. It explored the subjective experience, occurrence, and content of a significant mental image in each mood state, retrospectively. Each item is rated on a 7-point scale (from -3 “not at all” to 3 “extremely”), or 9-point scale (from 1 “not at all” to 9 “extremely”). Higher scores indicate greater emotional impact of the image.

#### Cognitive (Non-Emotional) Stages of Mental Imagery

##### Imagery Inspection

This is considered the third stage of cognitive mental imagery (1) and can be assessed with the Letter Corner Classification Task (LCCT) (64). This task involves assessing the object-based spatial characteristics of four block capital letters (F, N, Z, G). Each letter was marked with an asterisk in the bottom left corner, and an arrow travelling clockwise around the letter. The task was split into three stages, each involving the letters being presented, which participants were asked to memorize, followed by the letters being removed from sight. In stage one, participants were then asked to reproduce each letter, starting at the asterisk and moving clockwise. In stage two, participants were asked to categorize the corners of each letter as “top” or “bottom” by moving clockwise around the letter, starting from the asterisk and indicating “yes” if the corner is at the extreme top or bottom of the letter, and indicating “no” otherwise. In stage three, participants were asked to categorize the corners of each letter as “outside” or “inside”, by moving clockwise around the letter, starting from the asterisk and indicating “yes” if the corner is at the extreme left or right of the letter, and indicating “no” otherwise. Error rates and response times were recorded.

##### Imagery Manipulation

This is considered the fourth/final stage of cognitive mental imagery (1) and can be assessed with the Mental Rotation Task (MRT) (65). In a computerized version of the MRT, participants were shown pairs of three-dimensional line drawings and asked to decide whether the drawings were of the same rotated object or of different objects. Participants completed a practice trial followed by three progressively more difficult levels of transformation, based on the angular disparity between the two shapes (easy: 50; medium: 100; difficult: 150). Error rates and response times per difficulty level were recorded. Two performance parameters were derived based on response times (41, 66): i) the intercept-index, deemed to represent the sensory-motor component of task response, and ii) the slope-index, deemed to represent the spatial-ability (imagery-based) component of task response.

### Statistical Analyses

Outliers above or below three standard deviations (*SDs*) of the mean were excluded. Groups were compared on baseline and clinical characteristics and on key outcomes from each imagery assessment based on previous literature (32). Each continuous dependent variable was examined with mixed-model analysis of variance with groups as fixed factors and modeling random effects for twin pairs to account for dependence within these. Categorical variable (sex only) was examined with logistic regression with groups as predictors and within twin-pair dependence adjusted for using generalized estimating equations estimates of the standard errors.

For our primary analyses, we included our three key groups, i.e., affected in remission (combining BD and UD), high-risk and low-risk, followed by pairwise comparisons when relevant. Comparing the affected (remitted) group with the low-risk group would help determine whether and which imagery abnormalities persist in remission; comparing the high-risk group with the other two groups would help determine whether and which imagery abnormalities are present in individuals who have not developed BD/UD despite genetic liability (consistent with the notion of an endophenotype for affective disorders). Effect sizes as Cohen *d* (67) were included to aid interpretations (0.30 = small; 0.50 = medium; 0.80 = large).

We conducted three additional sets of secondary (exploratory) analyses. First, we repeated the above analyses with only BD or UD on selected imagery measures for which previous literature indicates discrepancy between disorders (and hence combining both groups may not be always appropriate): i) emotional imagery of positive valence (PIT and MII) may be enhanced in BD only (35, 68, 69) and ii) aspects of mental rotation performance may be affected in BD (32) and UD (41). Second, we directly contrasted the BD and UD affected (remitted) groups, as previous studies have contrasted imagery measures between BD and UD groups predominantly during depression illness at the time of assessment (32). Finally, we conducted a series of multiple linear regressions with baseline clinical characteristics (HDRS and STAI-Y) as predictors, and key imagery outcomes (emerging as significant from our primary analyses) as dependent variables, based on our previous research demonstrating associations between emotional imagery and symptoms transdiagnostically (32). Manic symptoms (YMRS) were not included as these were matched between groups (see Results). Initially both predictors were entered simultaneously, and then non-significant predictors were removed from the model stepwise until only significant predictors remained.

Significance level was set to alpha = .05 for two-sided hypothesis-testing (unless otherwise stated for directional hypothesis-testing). As our primary analyses involved multiple comparisons, we also applied the Benjamini-Hochberg procedure (70) to control for family-wise false discovery rate (*q* = .10), and we reported these when they changed the pattern of results (for one pairwise comparison only). Data analyses were conducted using SPSS (71).

## Results

### Baseline and Clinical Characteristics

Means and SDs are presented in **Table 1**. There were no significant group differences in sex, age, education, or IQ. As expected, the affected twins scored higher (than high-risk and low-risk groups) on baseline symptoms of depression and anxiety, but there were no significant differences between high-risk and low-risk twins. There were no significant group differences in symptoms of mania. A high proportion of affected twins in remission reported current pharmacological medications (**Table 1**). Restricting our primary analyses to twins without medication did not change the pattern of results, hence we report results including all twins.

**Table 1:** Baseline and clinical characteristics of affected, high-risk, and low-risk monozygotic twins for affective disorders.

	Affected (BD+UD; remitted)			High-risk			Low-risk			Overall group difference		
	*M*	*SD*	*n*	*M*	*SD*	*n*	*M*	*SD*	*n*	*F*	*df*	*p*
Age (years)	36.10	8.83	115	36.94	9.58	49	37.07	9.18	40	< 1	2, 98.94	.868
Education (years)	14.53	3.26	115	15.69	3.14	49	15.50	2.61	40	2.14	2, 107.06	.123
IQ	113.53	6.36	109	112.44	6.74	48	114.04	5.69	40	1.36	2, 110.87	.261
HDRS-17	4.84^a^	3.55	115	2.73^b^	2.46	49	1.89^b^	2.11	40	16.24	2, 114.35	< .001
STAI-state	31.81^a^	7.54	115	28.78^b^	6.78	49	26.98^b^	6.90	40	7.59	2, 170.98	.001
YMRS	1.83	2.13	115	1.51	1.31	49	1.25	1.53	40	1.54	2, 184.61	.217
	***n***	**%**		***n***	**%**		***n***	**%**				
Sex (female)	82	71		33	67		32	80		Wald chi-square (2, *N =* 204) = 1.30, *p =* .522		
Medication (yes)	63	55		3	6		0					
Antidepressant	45	39		1	2		0					
Mood stabilizer	22	19		0			0					
Antipsychotic	18	16		0			0					

BD, bipolar disorder; UD, unipolar disorder; HDRS-17, Hamilton Depression Rating Scale; IQ STAI, State-Trait Anxiety Inventory; YMRS, Young Mania RatingScale.

### Subjective Domains of Mental Imagery

#### Spontaneous Imagery Use

Overall group difference in SUIS scores was not statistically significant (**Table 2**), indicating an absence of between-group differences in spontaneous use of non-emotional imagery in everyday life.

**Table 2:** Subjective domains of mental imagery in affected, high-risk, and low-risk monozygotic twins for affective disorders.

	Affected (BD+UD; remitted)			High-risk			Low-risk			Overall group difference			Effect size (d) of pairwise comparisons		
	*M*	*SD*	*n*	*M*	*SD*	*n*	*M*	*SD*	*n*	*F*	*df*	*p*	*AF vs. LR*	*AF vs. HR*	*HR vs. LR*
**SUIS**	37.25	9.70	104	36.81	9.55	42	36.30	7.20	37	<1	2, 139.39	.942	0.18	0.08	0.08
**IFES**	33.48	15.37	88	25.21	13.88	38	19.00	10.61	32	11.46	2, 137.19	<.001	1.11	.68	.52
**PIT**															
Negative															
*Vividness*	2.70^a^	0.84	91	2.26^b^	0.85	38	2.17^b^	0.90	36	5.74	2, 156.05	.004	0.79	0.69	0.13
*Likelihood*	2.86^a^	0.49	89	2.52^b^	0.61	36	2.61^b^	0.54	35	5.77	2, 150.02	.004	0.62	0.84	0.19
*Experiencing*	2.66^a^	0.77	90	2.25^b^	0.90	38	1.97^b^	0.65	35	10.62	2, 147.09	<.001	1.23	0.71	0.41
Positive															
*Vividness*	3.43	0.96	87	3.68	1.16	39	3.50	1.00	34	<1	2, 144.89	.381	0.10	0.31	0.22
*Likelihood*	2.87^a^	0.75	86	3.33	0.67	36	3.17^b^	0.64	34	4.42	2, 143.80	.014	0.56	0.80	0.32
*Experiencing*	3.19	0.86	87	3.43	0.87	37	3.24	0.90	34	1.07	2, 146.95	.345	0.08	0.35	0.30
**MII**															
Low mood															
*Frequency*	4.58	2.39	95	5.05	2.28	40	4.86	2.14	28	<1	2, 139.76	.566	0.14	0.26	0.11
*Realness*	6.15	1.86	75	5.71	2.32	35	5.61	1.70	23	<1	2, 121.17	.381	0.42	0.30	0.07
*Compellingness*	6.21	2.04	76	5.64	2.33	36	5.87	1.58	23	1.00	2, 132	.369	0.24	0.38	0.14
*Demotivating*	6.64	2.49	74	5.18	3.01	34	4.58	2.50	24	7.15	2, 129	.001	1.01	0.78	0.23
Anxious mood															
*Frequency*	4.76	2.96	76	5.26	3.02	31	6.32	2.16	19	1.75	2, 107.53	.178	0.76	0.20	0.55
*Realness*	6.63	1.80	57	6.75	2.07	24	6.00	2.29	19	<1	2, 97	.403	0.46	0.09	0.49
*Compellingness*	6.63	2.10	57	6.5	1.93	24	6.32	1.95	19	<1	2, 95.66	.831	0.21	0.08	0.13
*Threatening*	6.57	2.43	56	6.17	2.81	24	5.53	2.76	19	1.18	2, 96	.311	0.56	0.22	0.31
High mood															
*Frequency*	5.76	2.43	100	5.95	2.43	44	5.90	2.01	31	<1	2, 151.13	.845	0.07	0.10	0.03
*Realness*	6.74	1.71	90	6.43	1.81	40	6.55	1.35	29	<1	2, 156	.585	0.16	0.25	0.10
*Compellingness*	6.73	1.67	90	6.55	1.50	40	6.57	1.50	30	<1	2, 137.80	.830	0.10	0.15	0.02
*Exciting*	7.51	1.51	90	7.45	1.20	38	7.81	0.96	27	<1	2, 141.09	.549	0.29	0.06	0.46

BD, bipolar disorder; UD, unipolar disorder; AF, affected group; HR, high-risk group; LR, low-risk group; SUIS, Spontaneous Use of Imagery Scale; PIT, Prospective Imagery Task; MII, Mental Imagery Interview.

Different subscripts (a, b) indicate significant differences after adjusting for false discovery rate.

The sample size per group differs by outcome due to missing data or outlier removal.

#### Emotional Mental Imagery

For the *IFES* (assessing the impact of intrusive future imagery), the overall group difference in total scores was statistically significant (**Table 2**). Affected twins in remission had higher IFES scores (indicating higher impact of intrusive prospective imagery) both relative to low-risk twins (*F*_(1, 89.93)_ = 8.60, *p = .*004), and to high-risk twins (*F*_(1, 85.70)_ = 15.49, *p* < .001). Low- and high-risk twins did not differ significantly in IFES scores (*F*_(1, 52.28)_ = 2.77, *p = .*102).

For the *PIT* (assessing the impact of deliberately-generated future imagery), overall statistically significant group differences were consistently found in response to imagined *negative* future scenarios (**Table 2**). Affected twins in remission rated negative future scenarios as more *vivid* than low-risk twins (*F*_(1, 72,58)_ = 7.64, *p = .*007), and also than high-risk twins (*F*_(1, 98.21)_ = 6.54, *p= .*012). Affected twins in remission rated these scenarios also as more *likely* to occur to them than low-risk twins (*F*_(1, 70.70)_ = 4.70, *p= .*034), and also than high-risk twins (*F*_(1, 91.31)_ = 10.05, *p = .*002). Finally, affected twins in remission reported “*experiencing*” these scenarios while imagining them, more so than low-risk twins (*F*_(1, 55.08)_ = 19.16, *p* < .001), and also than high-risk twins, (*F*_(1, 126)_ = 6.91, *p = .*010). The low- and the high-risk twins did not statistically significantly differ in ratings of vividness, likelihood (*F*’s <1), or “experiencing” (*F*_(1, 41.10)_ = 1.93, *p = .*173).

In contrast, overall statistically significant group differences in response to deliberately-generated imagery of *positive* future scenarios were found for ratings of *likelihood* only, but not for ratings of vividness or “experiencing” (**Table 2**). Pairwise comparisons showed affected twins in remission rated positive future scenarios as *less likely* to occur to them than low-risk twins (*F*_(1, 63.1)_ = 3.74, *p = .*058), and also than high-risk twins (*F*_(1,94.66)_= 6.90, *p = .*010), but there were no statistically significant differences in the latter two groups (*F* < 1). When controlling for false discovery rate, the latter pairwise comparison between affected twins and low-risk twins was no longer significant.

In the *MII* for low-mood states, there were no statistically significant overall group differences in time spent thinking in images (frequency), nor in ratings of images as “real” and “compelling” (**Table 2**). However, there were statistically significant group differences in how “demotivating” the image was. Affected twins in remission rated finding their images during low-mood more demotivating low-risk twins (*F*_(1,69.05) =_ 11.65, *p= .*001), and also than high-risk twins (*F*_(1,106) =_ 6.99, *p = .*009), but there were no significant differences in the latter two groups (*F*< 1). For images during anxious-mood and high-mood states, there were no statistically significant overall group differences in time spent thinking in images (frequency), nor ratings of other phenomenological properties of the images (**Table 2**).

To assess whether significant group differences in emotional imagery were driven by affected twins in remission reporting higher levels of residual depressive symptoms even during remission (**Table 1**), we repeated the above analyses with statistically significant findings without participants reporting scores > 8 in HDRS (threshold for full remission) (50), but the same pattern of findings remained (data not shown).

### Cognitive (Non-Emotional) Stages of Mental Imagery

#### Letter Corner Classification Task

The overall group difference in LCCT performance was not statistically significant for both error rates and reaction times (**Table 3**).

**Table 3:** Cognitive (non-emotional) domains of mental imagery in affected, high-risk, and low-risk monozygotic twins for affective disorders.

	Affected (BD+UD; remitted)			High-risk			Low-risk			Overall group difference			Effect size (d) of pairwise comparisons		
	*M*	*SD*	*n*	*M*	*SD*	*n*	*M*	*SD*	*n*	*F*	*df*	*p*	*AF vs. LR*	*AF vs. HR*	*HR vs. LR*
**LCCT**															
*Error rates*	9.42	9.98	98	10.3	9.90	43	8.27	8.23	33	0.54	2,87.05	0.586	0.13	0.09	0.24
*RT (msec)*	12.18	5.42	98	12.09	4.45	43	10.17	3.72	33	1.68	2,106.87	0.191	0.44	0.02	0.46
**MRT**															
*RT easy (msec)*	3,152.46	551.22	92	3,187.32	459.00	45	3,369.02	60.30	31	1.84	2,110.72	0.164	0.46	0.08	0.46
*RT medium (msec)*	3,470.64	509.95	92	3,426.17	463.43	45	3,592.08	511.48	31	0.80	2,117.89	0.453	0.42	0.10	0.44
*RT difficult (msec)*	3,558.59	472.36	92	3,562.08	447.92	45	3,705.57	459.01	31	1.16	2,133.55	0.315	0.40	0.01	0.43
*Intercept (msec)*	2,982.37	624.59	92	3,015.37	506.15	45	3,241.45	667.87	31	1.70	2,111.26	0.187	0.48	0.08	0.49
*slope*	207.20	160.10	92	171.98	214.39	45	167.93	169.56	31	0.86	2,143.23	0.424	0.34	0.25	0.03
*errors*	24.32	14.78	92	20.49	11.22	45	24.62	11.05	31	2.13	2,100.33	0.124	0.02	0.31	0.41

BD, bipolar disorder; UD, unipolar disorder; AF, affected group; HR, high-risk group; LR, low-risk group; LCCT, Letter Corner Classification Task; MRT, Mental Rotation Task; RT, response time.

The sample size per group differs by outcome due to missing data or outlier removal.

#### Mental Rotation Task

The overall group difference in MRT performance was not statistically significant, for the intercept (index of sensory-motor processing), slope (index of spatial/imagery processing), nor error rates (**Table 3**).

### Secondary Analyses

#### Separate Bipolar Disorder/Unipolar Depression Analyses

For the analyses on the BD only (affected-BD, high-risk-BD, and low-risk), there were no overall statistically significant group differences in positive imagery (in PIT and MII) (*F*’s < 2.54, *p*’s > .090) or MRT performance (*F*’s < 1.45, *p*’s > .246). Similarly, for the analyses on the UD only (affected-UD, high-risk-UD, and low-risk), there were again no statistically significant overall difference in positive imagery (*F*’s < 2.65, *p*’s > .075) or MRT performance (*F*’s < 2.07, *p*’s > .132).

#### Bipolar Disorder ***Vs***. Unipolar Depression Within Affected Twins

We compared BD *vs*. UD twins in remission on all our key subjective and cognitive imagery outcomes. There was only statistically significant group difference in one outcome of the MII. Specifically, affected-BD twins in remission rated their significant image during low-mood states as more “compelling” (*M =* 7.06, *SD* = 1.73) than affected-UD twins in remission (*M =* 5.95, *SD* = 2.06; *F* *_(_*_1,74)_ = 4.24, *p = .*043).

#### Clinical Characteristics Across the Full Sample

Across all groups, higher levels of depressive symptoms were significantly associated with i) higher IFES scores; ii) rating *negative* imagined scenarios in the PIT as more vivid, more likely to occur and accompanied with more feelings of “experiencing”; iii) rating *positive* imagined scenarios in the PIT as less likely to occur to them; and iv) reporting (in the MII) that images during low-mood states are more “demotivating”. Additionally, higher levels of anxiety symptoms were significantly associated with i) higher IFES scores; and ii) rating *positive* imagined scenarios in the PIT as less likely to occur to them.

## Discussion

### Main Findings

This large study of 204 monozygotic twins investigated—for the first time—whether mental imagery abnormalities are present in i) affected twins with affective disorders in remission and ii) twins with high familial risk for affective disorders. Regarding the first aim, we found that remitted twins with a history of affective disorders reported greater emotional impact of intrusive prospective imagery, and greater vividness, likelihood, and subjective experience of (deliberately-generated) negative prospective imagery compared with unaffected twins at either low-risk or high-risk of affective disorders. That is, affected twins are more prone to imagining the future (whether a desired holiday or a feared examination) and when they did so it felt more real and emotional. The affected twins in remission also reported less likelihood of (deliberately-generated) positive prospective imagery compared to both high-risk and low-risk twins (however, the latter result did not survive correction for multiple comparisons). In contrast, the twin groups did not differ on measures of cognitive stages (non-emotional) of mental imagery.

In relation to the second aim, we found no evidence of mental imagery abnormalities in twins with high familial risk for affective disorders but who were nevertheless not affected. Finally, mood and anxiety symptoms were significantly associated with greater emotional impact of prospective imagery across our entire sample, replicating findings that support a dimensional and transdiagnostic role of imagery abnormalities in psychopathology (32).

### Theoretical Implications

Our findings extend previous work identifying abnormalities in prospective emotional mental imagery across affective disorders in the presence of significant depressive symptoms (7, 32, 33, 37). Interestingly, our pattern of results were the same for analyses conducted using a sub-sample restricting the affected group in remission to only those individuals with no residual subclinical symptomatology (HDRS scores <8; *n =* 89). This suggests that imagery abnormalities are not just related to residual affective symptomatology—these critically may represent a trait-related phenomenon in individuals with affective disorders. The distinction between “state” or “trait” needs to be further delineated, for example using direct comparisons of participants during remission versus during illness episodes (while acutely depressed and/or in manic/mixed episodes). While imagery-based abnormalities that are equivalent during both illness and remission are consistent with the notion of “trait” markers, those abnormalities that more pronounced during illness episodes may additionally represent “state” markers.

The association between biases in prospection and psychopathology remains understudied, although recent research suggests its potential relevance for depression (72) and anxiety (73). Our study indicates, for the first time, that individuals with a history of affective disorders continue to experience vivid intense images of *negative* future events even during long-term remission, similar to other cognitive-emotional biases including imagery of the past (27). Instead, abnormalities in *positive* prospective imagery appeared limited to *likelihood* ratings across affective disorders (although these results did not survive more stringent corrections for multiple comparisons), and did not differentiate BD *versus* UD (both in remitted states). Future replications need to confirm if the inability to experience vivid *positive* images of future events as previously reported is only prominent during illness/depressive states (63, 74).

Our findings also highlight that unlike emotional imagery, cognitive (non-emotional) imagery processes appear intact across affective disorders in remission. While we previously found some evidence of both better and worse performance on cognitive stages of imagery in a sample of mixed euthymic and depressed BD (32), the present study confirms that cognitive stages of imagery remain largely preserved in affective disorders (32). These cognitive processes rely on general executive function as well as on more specific visuospatial abilities (1). How these processes—as measured by cognitive imagery tasks—compare to other components and measures of cognition remains unclear. This is relevant as executive function in particular is often compromised in recovered individuals with affective disorders (75, 76). However, affective disorders are largely heterogeneous with regards to cognition (77, 78); for instance, affected twins in our sample did not show any cognitive deficits compared to low-risk twins (54). Answering the above question may be relevant with a view to personalizing therapeutic interventions. As examples, common cognitive therapy techniques such as reappraisal may rely on well-functioning cognition and hence be less efficacious if executive function is compromised, and likewise, imagery-based techniques could be advantageous in those with intact cognitive stages of imagery.

Abnormalities in prospective imagery were not present in high-risk twins compared to low-risk twins, indicating that these do not fulfill the criteria for an illness “endophenotype” (28). Our data are more consistent with the notion that imagery-based abnormalities reflect (neuro)cognitive markers of the disorder itself—abnormalities that are likely to play a role in increasing future relapse given their persistence in remitted states. We note that previous research has shown altered emotional mental imagery in individuals with *phenotypic* (rather than genetic/familial) risk for BD, mainly based on the presence of hypomanic-like experiences (32, 43, 69, 79). However, our study was the first to focus instead on genetic-based risk using a twin design enabled by the unique Danish registers (49). As a potential explanation for this discrepancy, maladaptive prospective emotional imagery may be associated with phenotypic characteristics of affective disorders that lie on the same dimension of clinical symptoms, such as the actual presence of dysphoric mood (47, 62) or hypomanic-like experiences (32, 43, 69, 79), but we did not measure such features in our high-risk twins. Another potential explanation is that our high-risk twins had an average higher age compared to at-risk groups from previous research. Thus it is also possible that our sample included individuals who had a familial risk but were actually “resilient” to developing affective disorders, at least in terms of mental imagery characteristics. We have previously argued that higher extroversion and lower neuroticism may mediate sensitivity to adverse events and better functioning in this high-risk sample (56), although only future longitudinal studies will be able to clarify factors determining vulnerability or resilience.

Notably, we found no evidence of differences between affective disorders categories of BD *vs*. UD. The only exception was that remitted BD twins retrospectively described their imagery at times of depressed mood as more compelling compared to remitted UD twins. This is consistent with previous findings on suicidal imagery (37), suggesting that when asking about past mental images during depressive episodes it may be important to distinguish for specific suicidal content to clarify potential phenomenological differences. We also found that greater emotional impact of prospective imagery was associated with anxiety and low mood symptoms across our whole sample. If replicated in samples with a larger number of individuals with BD, overall these results will indicate that individuals with affective disorders may share a trait-like common profile of mental imagery characteristics, in line with a most recent dimensional and transdiagnostic model of the role of emotional imagery in psychopathology (32).

### Clinical Implications

If prospective emotional imagery remains abnormally enhanced after recovery from affective disorders episodes, this could represent a vulnerability factor for future relapses through their impact on emotion and mood instability. We have previously proposed that vivid intense mental images may act as an emotional amplifier and maintain mood instability (7, 29). These novel findings highlight the importance of future longitudinal studies investigating whether imagery abnormalities during remission indeed predict subsequent illness recurrence rates.

Prospection has a key functional role in individuals’ daily life: we imagine future scenarios as a way to plan action, anticipate potential events and direct decision-making, manage uncertainty, and strengthen motivation toward goals (30, 31). Future imagery can also support emotion regulation, e.g., by anticipating future rewards to overcome present difficulties (80, 81). If prospection is biased in those with past affective episodes, remitted individuals will experience negative future scenarios more vividly, more likely to happen, and such intrusive images of the future will carry maladaptive emotional impact.

Finally, our findings indicate that imagery-based treatment innovation could be guided toward tertiary rather than primary prevention—at least in relation to *familial* risk. Building on previous therapy protocols (82–84) and recent experimental studies (85), targeting prospective imagery abnormalities using imagery-based cognitive therapy techniques has potential as a brief relapse prevention intervention, in particular in patients with greater mood instability (86). Emerging work in cognitive science also suggests that simple competing tasks can dampen recurrent “flashforward” imagery by taxing working memory while holding the flashforward image in mind (15, 87). Future studies should measure change in prospective imagery characteristics after imagery-based interventions to develop novel interventions based on mechanistic hypotheses (16).

### Limitations and Strengths

Several limitations should be noted in our study. First, we did not include a dizygotic twins group; hence, we could not assess the interaction between environmental and genetic influence on variance, which is preferable when investigating potential endophenotypes. Second, we did not directly compared twins at high-risk for UD *versus* at high-risk for BD. Such comparison could establish whether familial risk in imagery abnormalities is more evident in high-risk twins given a co-twin history of BD rather than UD, because of the higher heritability of BD (19) relative to UD (20). Unfortunately we had low power to run those analyses because the high-risk for BD group was very small (*n* = 11). Third, we did not include a full comprehensive assessment of mental imagery (40), so it is possible that biases in other imagery domains—such as trauma memory imagery (5, 14, 88) and/or reward/motivational imagery (89, 90)—are also present in remitted affective disorders and/or high-risk twins. This might be relevant to the prevalence of trauma in affective disorders (91) and of persistent anhedonic symptomatology (92), which are both associated with relapse and worse prognosis. Finally, results from our analyses comparing patients affected with UD *versus* BD (*n* = 84 *versus n* = 31, respectively) and from our secondary analysis should be interpreted with caution, given the small sample size of the separate BD group (*n* = 31 affected and *n* = 11 high risk, respectively).

Major strengths of our study are the large and population-based twin sample (enabling a unique design to test hypotheses regarding familial risk), twin groups well-matched on demographic variables, and the use of validated scales and tasks that allow building on previous research and future replication. Further, participants had been in long-term remission providing greater confidence that findings were not state-related (although it remains possible that imagery is also linked to states). Importantly, our findings are unlikely to be attributed to other cognitive confounders, as our groups did not differ in general cognitive function (54). Finally, to our knowledge, this was also the second study ever to combine emotional in tandem with cognitive (non-emotional) testing of imagery abnormalities in a clinical sample.

## Conclusions

Our study was the first investigation of mental imagery characteristics in affective disorders using a large population-based monozygotic twin sample. For the first time, we show that abnormalities in emotional prospective imagery also persist after recovery from acute episodes, but are not present in unaffected individuals at familial risk, suggesting that imagery characteristics are unlikely to fulfill the “endophenotype” criterion. Thus, mechanistically, imagery-abnormalities in affective disorders (BD and UD) may be best conceptualized as cognitive markers of the disorders, contributing to both psychopathology maintenance and possibly future relapse. Our findings also highlight that emotional imagery phenomenology can be useable in clinical practice (93), as a hallmark of psychopathology and ongoing vulnerability. Abnormalities in prospective emotional imagery represent a promising target for mechanism-testing in treatment innovation in affective disturbances.

## Data Availability Statement

The raw data supporting the conclusions of this manuscript will be made available by the authors, without undue reservation, to any qualified researcher.

## Ethics Statement

The study was approved by the local ethics committee (H-3-2014-003) and the Danish data protection agency (2014–331–0751). The patients/participants provided their written informed consent to participate in this study.

## Author Contributions

MS contributed to study design, data analysis, data interpretation, and manuscript writing. AL-Z conducted data analysis and contributed to data interpretation and manuscript writing. PT contributed to data processing, data analysis, and manuscript writing. IM conducted data collection and contributed to study design and data processing. LK and MV contributed to study design. EH contributed to study design, data interpretation, and manuscript writing. MV designed, obtained the ethical permissions, led the register linkage, and supervised together with LK the recruitment of participants and data collection. KM designed and managed the study, including data collection and processing, and contributed to manuscript writing. All authors reviewed and approved the finalmanuscript.

## Funding

MD was funded by the United Kingdom Medical Research Council intramural programme to EH (MRC-A060-5PR50). IM was supported by The Lundbeck Foundation (grant number R108-A10015) and the Hørslev Foundation. EH was supported by The Wellcome Trust (WT088217), the Lupina Foundation, and the Swedish Research Council (2017–00957). KM is supported by The Lundbeck Foundation and Weimann Foundation.

## Conflict of Interest

MS and EH are co-authors of a book on imagery-based cognitive therapy (Guildford Press, 2019). LK and MV have within recent 3 years been consultants for Lundbeck.

The remaining authors declare that the research was conducted in the absence of any commercial or financial relationships that could be construed as a potential conflict of interest.
